# Effect of Physical Modifications on Physicochemical and Functional Properties of Walnut Protein

**DOI:** 10.3390/foods12193709

**Published:** 2023-10-09

**Authors:** Shanshan Li, Zhe Liu, Xue Hei, Chao Wu, Xiaojie Ma, Hui Hu, Bo Jiao, Jinjin Zhu, Benu Adhikari, Qiang Wang, Aimin Shi

**Affiliations:** 1 Institute of Food Science and Technology, Chinese Academy of Agricultural Sciences, Key Laboratory of Agro-Products Processing, Ministry of Agriculture and Rural Affairs, Beijing 100193, China; lss05112021@163.com (S.L.); wangqiang06@caas.cn (Q.W.); 2 School of Science, RMIT University, Melbourne, VIC 3083, Australia

**Keywords:** walnut protein, physical modification, physicochemical properties, functional properties

## Abstract

Walnut protein is a high-quality vegetable protein with promising applications in the food industry; however, its potential is hindered by low solubility and associated properties. We utilized various physical modification techniques (cold plasma; ball milling; superfine grinding; ultrasound; wet ball milling; and high-pressure microjet) to enhance walnut proteins’ physicochemical and functional properties. The changes in particle size, microstructure, surface hydrophobicity, fluorescence, solubility, foaming, and emulsification were investigated. Cold plasma and ultrasound treatments minimally affected particle size and morphology. Cold plasma increased the particle size D_4,3_ from 145.20 μm to 152.50 μm. Ultrasonication reduced the particle size D_4,3_ to 138.00 μm. The variation was within ±10 μm, while the particle size of walnut protein significantly decreased after the other four modification treatments. The greatest variation in particle size was in the superfine grinding, with the D_4,3_ being reduced to 23.80 μm. Ultrasound treatment converted the β-sheet into an α-helix, while the other methods transformed the α-helix into a β-sheet. The dispersion stability notably improved after wet ball milling and high-pressure microjet treatments, which was accompanied by a significant increase in solubility from 6.9% (control) to 13.6% (wet ball milling) and 31.7% (high-pressure microjet). The foaming and emulsification properties were also enhanced through these modifications (foaming improved from 47% to 55.33% and emulsification improved from 4.32 m^2^/g to 8.27 m^2^/g). High-pressure microjet treatment proved most effective at improving solubility in the functional properties of walnut protein. These findings are expected to help broaden the potential utilization of walnut protein in the food industry, including in beverages and emulsions.

## 1. Introduction

Walnut protein is a valuable source of vegetable protein due to its richness in 18 amino acids, including 8 essential amino acids. The abundance of essential amino acids makes it highly desirable from a nutritional standpoint. Moreover, the natural abundance of walnut protein further contributes to its economic advantage. Walnut protein is primarily derived from walnut meal, which is a byproduct of walnut oil processing. Walnut meal is currently used as a low-cost ingredient in animal feed, and its potential or high value is not fully recognized [1,2]. This may be mainly due to the poor solubility of glutenin, which accounts for more than 70% of walnut protein [3,4]. Due to poor solubility, the functional properties of walnut protein, such as emulsification and foaming, are also inferior.

Chemical, enzymatic, and physical modifications are used to improve the functional properties of plant proteins, including that of walnut protein. Chemical modification utilizes chemical reagents to modify protein side chains, including the replacement of specific amino or hydroxyl groups in amino acid residues to alter the overall net charge [5,6,7]. Enzymatic modification utilizes enzymes that act on specific sites of protein and alter its structure [5]. Physical modification involves the application of a specific force field to modify the structure (confirmation) of the protein to achieve redistribution, unfolding, better dispersion, and solubility. In addition to traditional heat treatment, novel physical modification techniques such as ultrasound, homogenization, low-temperature plasma, ball milling, superfine grinding, and high-pressure microjet techniques are frequently used to modify proteins [8]. Physical modification techniques can be classified as dry modification or wet modification depending on the state of the sample during the modification process. Chemical modification is not preferred in the food industry due to the preference to ‘clean label’ products. Enzymatic modification also suffers from the problems of high enzyme cost, long processing time, and an uncontrollable hydrolysis. Relying solely on enzymatic modification may not sufficiently enhance the functional properties of walnut protein. In the case of walnut protein, physical modification might be preferred over chemical or enzymatic modification as it avoids the introduction of non-native chemicals, is cost-effective, and is suitable for industrial-scale production.

It was recently reported that plasma treatment reduced the average diameter of zein micelles and increased the solubility of zein in both neutral and acidic water solutions [9]. Sun et al. showed that ball milling of whey protein concentrates for 8 h using a high-energy nano ball mill resulted in changes in the secondary structure of the proteins, indicating that this level of ultra-micronization disrupted the intermolecular interactions [10]. Zhu et al. showed that ultrasonic treatment improved the water solubility, emulsification, and emulsion stability of walnut isolate protein [4]. It was also shown that walnut protein is modified by high speed shear, ultrasound, and high hydrostatic pressure; all of these improved the functional properties of walnut protein to some extent [11,12,13]. Most of the studies thus far have focused on determining the effects of a single modification technique (e.g., physical, chemical, or enzymatic) and have typically selected one physical modification technique among the various techniques mentioned above. Given the clear advantages of physical techniques, it is of practical interest to evaluate the effectiveness of readily available physical modification techniques to improve the physicochemical and functional properties of walnut protein. Improvements in these properties would pave the way for the high-value utilization of walnut protein.

This study investigated the effects of six physical modification techniques on the solubility and other functional properties of walnut protein. The dry techniques investigated included cold plasma, ball milling, and superfine grinding, while the wet techniques included ultrasound, wet ball milling, and a high-pressure microjet method. To understand the mechanism behind the improved functional properties, we employed various analytical methods, including particle size, SEM, surface hydrophobicity, intrinsic fluorescence, and FT-IR. The outcomes are expected to provide an effective and environmentally friendly strategy for enhancing the solubility of walnut protein, thereby adding value to this relatively underutilized protein.

## 2. Materials and Methods

### 2.1. Materials

Walnut protein (protein, moisture, and ash contents were 78.16%, 5.32%, and 1.72%, respectively) was obtained from Zeilan Biotech Co., Ltd. (Xi’an, China). Bovine serum albumin (BSA, standard grade) was purchased from Solarbio Inc. (Beijing, China). The 1-8-Anilino naphthalenesulfonate (ANS) was obtained from Cayman Chemical Company (Ann Arbor, MI, USA).

### 2.2. Preparation of Different Physically Modified Walnut Protein

Based on the preliminary experiments, we used three physical methods based on dry techniques: cold plasma (TS-VPL10-GT), dry ball milling (Retsch PM100), and superfine grinding (NETZSCH CGS 10). Three wet physical modifications were used: ultrasound (KQ-500DE), wet ball milling, and high-pressure micro-jet (Mini DeBEE type). The cold plasma method was used at 480 W for 5 min; the dry ball milling technique was used at 400 rpm for 10 min; and the superfine grinding approach was performed at 8000 rpm for 24 min. Three physical methods based on wet techniques were used at 8% (*w*/*v*); this walnut protein solution was subjected to ultrasound treatment at 400 W for 1 h, wet ball milling at 400 rpm for 1 h, and homogenized twice using the high-pressure microjet technique at 180 MPa.

### 2.3. Measurement of Size Distribution

The particle size and size distribution of walnut proteins were measured using a laser particle size analyzer (Master Sizer 3000, Malvern instruments, Worcestershire, UK) following the method of Liu et al. with slight modifications [14,15]. The samples were added to a stirred measuring cell containing 400 mL of deionized water until the obscuration reached 10–15%. The refractive indices of the deionized water and protein particles were 1.330 and 1.450.

### 2.4. Scanning Electron Microscopy

The microstructure of the physically modified samples was determined using a scanning electron microscope (SEM) (SU8010, Hitachi, Tokyo, Japan). By following and slightly modifying the methodology of Liu et al., all samples were prepared to a concentration of 0.1% and 5 µL of the solution was pipetted onto a silicon wafer and dried [14]. The solution was then evenly distributed on the conductive tape. Gold sputtering and imaging were performed using an ion sputtering apparatus under an argon atmosphere and 25 kV of electron accelerating voltage.

### 2.5. Surface Hydrophobicity

The hydrophobicity of the samples was measured in terms of the fluorescence intensity using a F-2500 fluorescence spectrophotometer (Hitachi, Japan) according to Qin et al. [12] with slight modifications. Seven groups of walnut protein sample solutions were prepared, each with a concentration of 1%, as described in Section 2.3. These samples were then centrifuged at 6000× *g* for 20 min at 25 °C. After centrifugation, the supernatant was collected to determine the protein content, for which the Lowry method was used. The collected supernatant was diluted with deionized water to achieve a concentration range of 0.04–0.5 mg/mL. To analyze the samples, 4 mL of each solution was mixed with 20 uL of 8 mM ANS solution and thoroughly shaken. The mixture was placed away from light for 10 min. The excitation and emission wavelengths of 390 nm and 470 nm were used in this analysis. The surface hydrophobicity (H0) was determined as the initial slope of the curve plotting fluorescence intensity (vertical axis) against protein concentration (horizontal axis).

### 2.6. Intrinsic Fluorescence

Intrinsic fluorescence emission spectra were measured according to Zhang et al. [16] with slight modifications, for which the fluorescence spectrophotometer was used. For this, 10 mg/mL of aqueous protein solution was prepared at pH 7.0, mixed thoroughly, and centrifuged at 6000× *g* for 15 min. The supernatant was collected and diluted to a concentration of 0.25 mg/mL for the determination of protein content using the Lowry method. The experimental conditions for these tests were as follows: excitation wavelength at 290 nm, scanning speed at 300 nm/s, and scanning range from 300 nm to 450 nm. Additionally, the width of the slit was maintained at 2.5 nm.

### 2.7. Fourier Transform Infrared Spectroscopy (FT-IR)

FT-IR analyses of protein samples were carried out using the attenuated total reflection (ATR) crystal surface using a spectrometer (TENSOR 27, Bruker, Saarbrucken, Germany). The method of Chen et al. was slightly modified by placing 1 mg/mL of protein solution on top of the ATR attachment [17]. A total of 64 scans were performed and averaged over a range of 500 to 4000 cm^−1^ using a resolution of 4 cm^−1^. The spectra of the amide I band (between 1700 and 1600 cm^−1^) were analyzed using PeakFitv 4.12 software. The position of each subpeak was determined relative to the secondary structure components to obtain protein secondary structure features.

### 2.8. Determination of the Stability of the Walnut Protein Solution

Seven sets of walnut protein sample solutions at 1% (*w*/*v*) concentration were prepared according to Li et al. [18] as described in Section 2.3, and the samples were placed in cylindrical glass vials and tested for their light scattering (multiple light scattering instrument, Toulouse, France). After a resting time of 10 min, the samples were scanned in the range of 2 to 45 mm, and the determination process took 2 h. The temperature in these tests was 25 °C, and a total of 31 scans were performed for each sample. A backscattered light (BS) curve of the Turbiscan stability profile was obtained and used to obtain the Turbiscan Stability Index (TSI).

### 2.9. Solubility

The solubility was measured according to Li et al. [18] with slight modifications. For these tests, solutions of unmodified and physically modified walnut protein following the dry route were prepared at 1% (*w*/*v*) with distilled water. The physically modified walnut protein following the wet route was diluted to 1% by mass. The samples were vortexed for 2 min and centrifuged at 6000× *g* for 10 min at 20 °C. The protein content in the supernatant was measured using the Lowry method, and the protein concentration was calculated using bovine serum protein (BSA) as the standard (A = 0.3121c + 0.0568, R^2^ = 0.995; c and A represent the walnut protein concentration and absorbance, respectively). Solubility was expressed as the protein content in the supernatant divided by the protein content in the starting solution.

### 2.10. Foaming Capacity and Foaming Stability

These two parameters were measured according to Mao and Hua [19] with slight modifications. We added 20 mL of 1% (*w*/*v*) walnut protein solution to a clear glass measuring cylinder. It was sheared at a high shear rate (10,000 rpm) for 2 min. The volumes of the protein solution and foam after shearing were recorded. The foaming property was measured as the ratio of the volume of solution foam after homogenization to the original volume (20 mL) (Equation (1)). The foam stability was determined as the ratio of the difference between the volume of the protein solution after homogenization for 15 min and the original volume, which was calculated as follows:

(1) $$FC\left(\%\right)=\frac{V_{1}-V_{0}}{V_{0}}\times 100\%$$

(2) $$FS\left(\%\right)=\frac{V_{2}-V_{0}}{V_{0}}\times 100\%$$

where *V*_0_ and *V*_1_ represent the volume before whipping and the volume after whipping, respectively; *V*_2_ represents the volume after storage for 15 min.

### 2.11. Emulsification Activity Index and Emulsion Stability Index

The emulsion activity index (EAI) and emulsion stability index (ESI) were measured according to Sun et al. [20] with slight modifications. The emulsion was prepared by mixing 9 mL of walnut protein emulsion solution at a 1% *w*/*v* concentration with 3 mL of sunflower oil and performing homogenization at 10,000 rpm. We quickly aspirated 50 µL from the bottom of the emulsion into 4950 µL of 0.1% SDS and shook the solution well. The absorbance (A_0_) was measured at 500 nm. After the emulsion was allowed to stand for 10 min, the absorbance (A_10_) was measured again in the same manner. The EAI and ESI were calculated using Equations (3) and (4), respectively.

(3) $$EAI\left(m^{2}/g\right)=2\times 2.303\times \frac{A_{0}\times N}{10000\times \varphi \times 1\times C}$$

(4) $$ESI\left(min\right)=\frac{A_{0}}{A_{0}-A_{10}}\times (t_{10}-t_{0})$$

where *A*_0_ is the initial absorbance; *N* is the dilution factor; *φ* is the volume fraction of the oil phase in the emulsion; *C* is the initial concentration of protein (g/mL); *A*_10_ is the absorbance after 10 min; and t_10_ − t_0_ is the time difference between the two measurement times.

### 2.12. Observation of the Morphology of Emulsions Stabilized by Walnut Protein

The microstructure of the freshly prepared emulsion was obtained using a confocal laser scanning microscope (CLSM) (TCS-SP8, Leica, Wetzlar, Germany). We took 1 mL of the emulsion and added it to the laser confocal disc, then added 10 μL of Nile red and Nile blue dye, respectively. After mixing thoroughly, staining was carried out for 30 min under the condition of avoiding light. The stained emulsion was placed on a carrier table, and the observations were made at excitation wavelengths of 488 nm and 633 nm [21].

### 2.13. Statistical Analysis

All experiments were carried out in triplicate except in the case of SEM and CLSM. The statistical evaluation was performed using analysis of variance (ANOVA) and Duncan’s tests with 95% confidence (*p* < 0.05). The results are expressed as the mean ± standard deviation (SD).

## 3. Results and Discussion

### 3.1. Impact of Physical Modifications on the Physicochemical Properties of Walnut Protein

#### 3.1.1. Size Distribution

**Figure 1:**
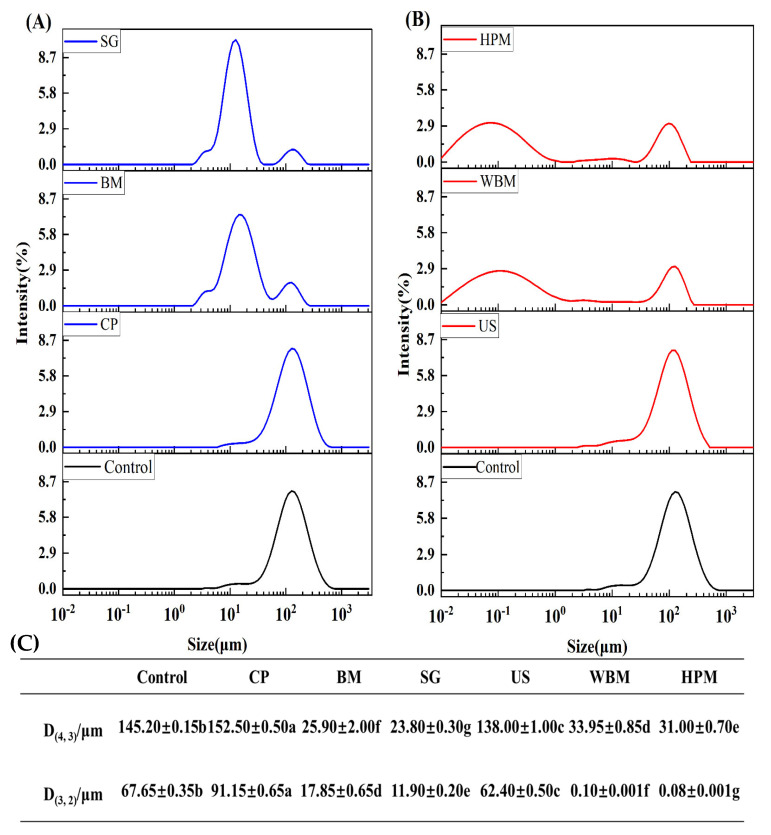
Particle size distribution of walnut protein after dry and wet routes of physical modifications ((**A**) Dry route of physical modification; (**B**) wet route of physical modification; (**C**) volume mean diameter (D_4,3_) and surface area mean diameter (D_3,2_)) (Control—control subjects; CP—cold plasma; BM—ball milling; SG—superfine grinding; US—ultrasound; WBM—wet ball milling; HPM—high-pressure microjet). (a–g) represent significant differences between walnut protein at the *p* < 0.05 level.

The particle size distribution of proteins affects the functional properties, including solubility. Therefore, the particle size distribution of the unmodified and six modified walnut protein solutions was determined (Figure 1). Cold plasma treatment led to an increase in protein particle size. Cold plasma released a large number of reactive ions with high energy, and when these high-energy particles bombard the protein surface, physical changes such as cross-linking and polymerization occur. Dry ball milling reduced the D_4, 3_ particle size of the proteins from 145.20 μm to 25.90 μm, which was probably due to the breaking of the proteins into smaller particles by the high-speed rotating milling balls within the walls of the jars. This is in agreement with the results of Liu et al. [22] who treated wheat gluten proteins using dry ball milling. The D_3,2_ particle size of walnut protein after wet ball milling was reduced from 67.65 μm to 100 nm, which indicates that particle size reduction using ball milling in the presence of water resulted in smaller particles. The change in particle size after superfine grinding treatment was similar to the results of dry ball milling, but superfine grinding was performed through air separation, shear, and other forms of crushing and thus achieves the purpose of particle size reduction. The particle size distribution peak of protein was slightly (insignificantly) shifted to the left after ultrasound. This indicates that the impact of the cavitation of ultrasound on the gluten of walnut protein is not significant [19]. The particle size and size distribution of walnut protein treated with the high-pressure microjet technique were similar to those obtained from wet ball milling. The high-pressure microjet method subjects protein molecules to cavitation, shear turbulence, and heating within a short period of time, and the agglomerates are broken into small particles, which ultimately affects the solubility and emulsification of protein [22,23,24]. Ball milling, wet ball milling, and high-pressure microjet modification significantly contributed to the reduction in particle size as well as the uniformity in size distribution.

#### 3.1.2. Microstructure

**Figure 2:**
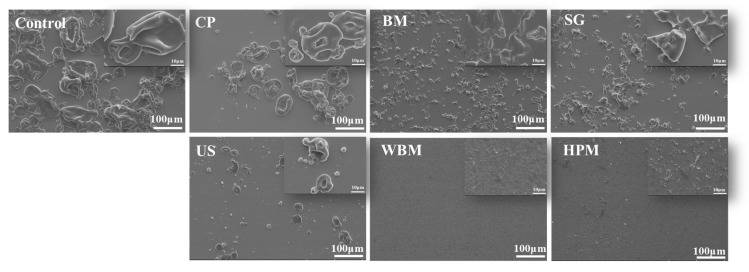
SEM images of walnut protein dry and wet routes of physical modifications (Control—control subjects; CP—cold plasma; BM—ball milling; SG—superfine grinding; US—ultrasound; WBM—wet ball milling; HPM—high-pressure microjet). (Magnification: large image—0.2 k; small image—2 k).

The microstructure of walnut protein after modification was observed using an SEM (Figure 2). Walnut protein without modification showed small spherical particles in an agglomerated condition. After dry treatment, the particles aggregated after cold plasma, generating fused particles with a larger particle size and rough surface, which agrees with the literature [9]. The globular structure of walnut protein particles was destroyed, and the morphology changed from spherical to flaky after ball milling and superfine grinding. In the wet modification treatment, the ultrasound preserved the initial spherical shape and morphology of the walnut protein. The high-pressure microjet and wet ball milling techniques destroyed the initial morphology of the walnut protein and created a particle of nanoscale size. This result indicates that the high-pressure microjet and wet ball milling techniques led to the breakdown and unfolding of insoluble aggregates, which in turn may have led to a looser protein structure. The solubility of protein molecules in aqueous systems is improved [25].

#### 3.1.3. Surface Hydrophobicity

The surface hydrophobicity (H_0_) of proteins influences their solubility and emulsifying properties. It refers to the antagonistic force between non-polar groups of protein and water molecules. This property plays an important role in the formation of tertiary protein structures through interactions among non-polar amino acid residues. Therefore, the surface hydrophobicity (H_0_) of samples was determined using the fluorescent probe (ANS), which specifically binds with the hydrophobic region of proteins. The H_0_ of walnut protein was significantly reduced after cold plasma and superfine grinding (Table 1). The reduction of H_0_ by cold plasma could be attributed to the bombardment of the walnut protein surface by high-energy particles that are generated during the treatment. This bombardment is believed to break the molecular chains, increase the oxygen-containing groups of the nucleoprotein, and expose polar groups, ultimately reducing the hydrophobic regions [26]. In the case of superfine grinding, proteins are subjected to high-intensity grinding and crushing forces, resulting in a significant reduction in particle size and an increase in the specific surface area. The non-polar bonding interactions of amino acid residues on the particle surface are weakened, leading to a decrease in hydrophobicity [27]. Interestingly, the surface hydrophobicity of wet-modified walnut proteins was significantly enhanced, probably due to the folding of the molecular structure, the disruption of internal chemical bonds, and the increased exposure of hydrophobic groups [28].

#### 3.1.4. Intrinsic Fluorescence

**Figure 3:**
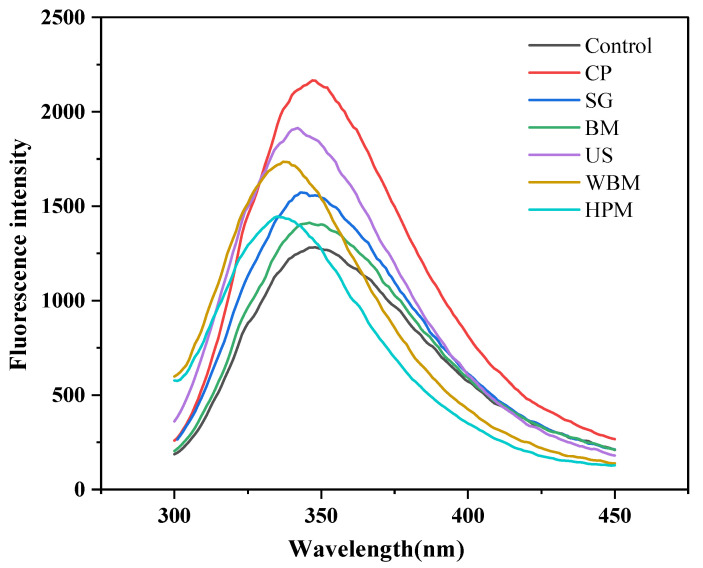
Intrinsic fluorescence spectra of walnut protein after dry and wet routes of physical modifications (Control—control subjects; CP—cold plasma; BM—ball milling; SG—superfine grinding; US—ultrasound; WBM—wet ball milling; HPM—high-pressure microjet).

The intrinsic fluorescence spectra of proteins originate from tryptophan (Trp). These spectra can respond to the changes in the tertiary structure of proteins by probing the hydrophilic and hydrophobic environment of tryptophan. The tryptophan-containing residues can fluoresce in the 300–400 nm range upon excitation at 290 nm [29]. As shown in Figure 3 and Table 1, the unmodified walnut protein showed the maximum tryptophan fluorescence intensity at 347.67 nm upon excitation at 290 nm. Except for cold plasma-modified walnut protein, which exhibited a blue shift in its maximum emission wavelengths, the physically modified walnut protein showed varying degrees of reduction in maximum emission wavelengths, indicating internal masking of tryptophan residues. The maximum fluorescence intensity of modified walnut proteins appeared to significantly increase. This may be due to a change in its aggregation state, thus altering the local environment of the molecule. These structural changes are considered to be responsible for the observed increase in fluorescence intensity.

#### 3.1.5. Secondary Structure

In the infrared spectrum, the secondary structure of a protein is reflected on the changes in the content or proportion of each subpeak in the amide I band (1600–1700 cm^−1^) [30]. The composition of the secondary structures of walnut protein before and after modification are shown in Table 2. The proportion of secondary structures of untreated walnut protein in descending order were α-helix, β-sheet, random coil, and β-turn. After the modification treatments, only the proportion of α-helix increased, and the proportion of β-sheet decreased after ultrasound modification, which is consistent with the impact of ultrasound on β-lactoglobulin [31]. The α-helix of walnut protein modified using other methods converted to β-sheet, which led to a decrease in α-helix and increase in β-sheet content. The α-helix structure is dominant in most native proteins [32]. Thus, these modification treatments disrupted the ordered structure of walnut protein and increased the disordered and loose structure. In the case of walnut protein modified by cold plasma, a higher proportion of α-helix converted to β-sheet and β-turn as a result of energetic particle bombardment. This observation is consistent with the results of peanut proteins treated with cold plasma by Ji et al. [33].

#### 3.1.6. Physical Stability in Aqueous Solution

**Figure 4:**
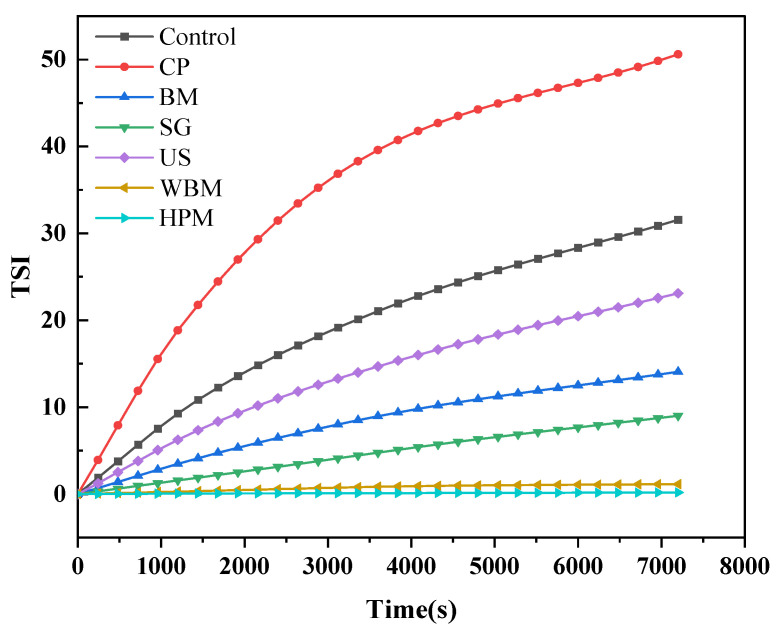
Variation of Turbiscan Stability Index (TSI) of walnut protein as a function of time after dry and wet routes of physical modifications. (Control—control subjects; CP—cold plasma; BM—ball milling; SG—superfine grinding; US—ultrasound; WBM—wet ball milling; HPM—high-pressure microjet).

Figure 4 shows the variation in the Turbiscan Stability Index (TSI) as a function of walnut protein subjected to dry (cold plasma, superfine grinding, ball milling) and wet routes of (ultrasound, wet ball milling, high-pressure microjet) physical modifications. A greater deviation from the starting line (TSI = 0) and a greater slope of the curve indicate a more unstable system [34]. The TSI indicated that the order of instability of the protein solution was: cold plasma > control > ultrasound > ball milling > superfine grinding > wet ball milling > high-pressure microjet. The stability of the protein solution became worse after cold plasma, indicating faster aggregation. Because the particle size data of cold plasma-treated walnut protein was not significantly different, its lowest stability was due to the change in its conformation and exposure of hydrophobic groups [35]. The best stability of protein solutions was observed in wet ball milling- and high-pressure microjet-modified walnut protein. This result indicated that these two wet modification methods significantly unfolded the protein structure and reduced the particle size, which improved the protein–water interaction and produced a more homogeneous and stable walnut protein solution [18].

### 3.2. Impact of Physical Modifications on the Functional Properties of Walnut Protein

#### 3.2.1. Solubility

**Figure 5:**
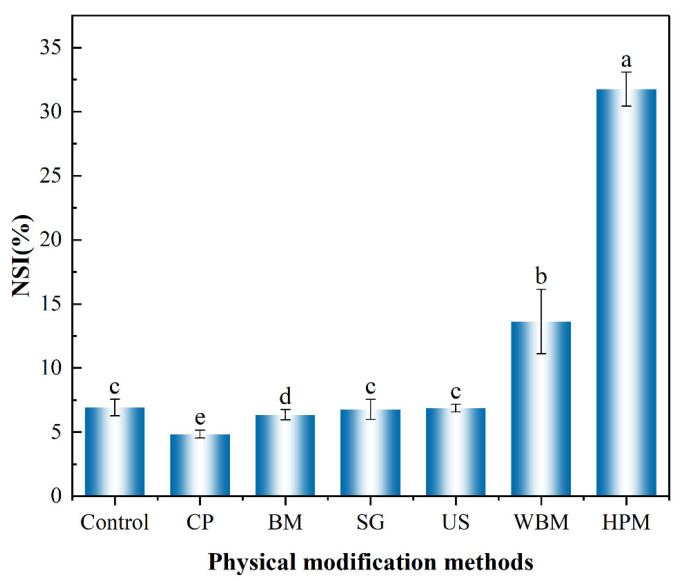
Solubility (in term of nitrogen solubility index, NSI) of walnut protein after dry and wet routes of physical modifications (Control—control subjects; CP—cold plasma; BM—ball milling; SG—superfine grinding; US—ultrasound; WBM—wet ball milling; HPM—high-pressure microjet); (a–e) represent significant differences between walnut protein at the *p* < 0.05 level.

The solubility of walnut protein is notably poor, mainly because more than 70% of it comprises insoluble glutenin. Other functional properties (e.g., foaming and emulsification) of walnut protein also depend on its solubility. The solubility changes of walnut protein after the physical modifications are shown in Figure 5 in terms of the nitrogen solubility index (NSI). After application of the dry route of modification, the solubility appeared to be slightly increased or even slightly decreased. In the case of modification by cold plasma, the solubility of the modified walnut protein decreased. This may be due to the fact that cold plasma treatment could release free radicals and reactive groups, which could increase protein aggregation [36]. The solubility of hemoglobin, pork gelatin, and bovine lung proteins was found to decrease after cold plasma treatment due to surface modification, protein–protein cross-linking, and aggregation [37]. It has been reported in some studies that the application of cold plasma increases the solubility of protein [27]. This inconsistency in findings may be due to the characteristics of the protein involved. The solubility of a protein is influenced by surface hydrophobicity, surface charge (zeta potential), and particle size [38,39]. There was no significant change in the solubility of walnut protein upon ultrasound treatment, which was consistent with its particle size data. Among all treatment methods tested, the highest solubility of walnut protein was obtained upon high-pressure microjet treatment, in which the NSI increased from 6.92% (unmodified) to 31.75% (modified). Under high shear and transient depressurization conditions, it is possible for the particle size of the protein to decrease and the surface charge to increase, both of which contribute to an increase in its solubility. It has also been shown that high-pressure microjet treatment breaks the intermolecular hydrogen bonds of proteins, loosening their chains and increasing their solubility in water [40].

#### 3.2.2. Foaming Properties

**Figure 6:**
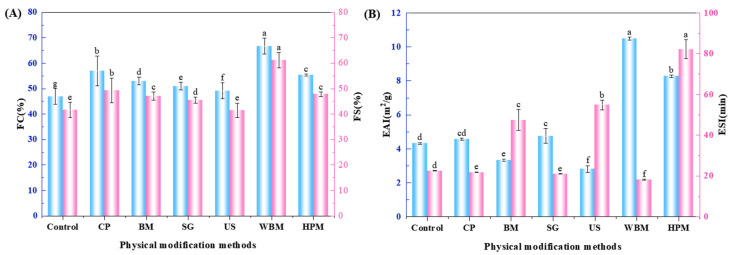
Foaming capacity (FC) and foam stability (FS) (**A**), emulsification activity index (EAI), and emulsification stability index (ESI) (**B**) of walnut proteins after dry and wet route of physical modifications (Control—control subjects; CP—cold plasma; BM—ball milling; SG—superfine grinding; US—ultrasound; WBM—wet ball milling; HPM—high-pressure microjet); (**A**)—(a–g) represent significant differences between walnut protein at the *p* < 0.05 level. (**B**)—(a–f) represent significant differences between emulsions at the *p* < 0.05 level.

Protein molecules are amphiphilic in nature and are capable of stabilizing air–water (foam) interfaces. The non-covalent interaction of proteins at the interface creates a film with low tension, which favors the formation and stability of foam [41]. From the results in Figure 6A, it can be seen that both foaming performance and foam stability were improved after modification, which could be attributed to the increase in the flexibility of the chain. The unfolding of proteins at the air–water interface is known to form a dense network structure, which improved the binding ability of proteins to air, thus improving their foam stability. For example, superfine grinding sufficiently reduced the size of protein particles and increased the specific surface area. This enabled the walnut protein particles to adsorb on the air–water interface and form a stable film. The foaming performance and foam stability were improved. [42]. In this regard, Qin et al. showed that high hydrostatic pressure treatment improved the foaming properties and foam stability of walnut protein [12].

#### 3.2.3. Emulsifying Properties

**Figure 7:**
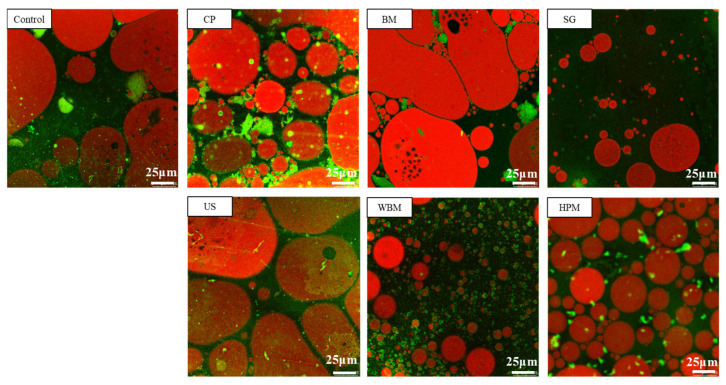
Microstructure of emulsions prepared from different physically modified walnut proteins (Control—control subjects; CP—cold plasma; BM—ball milling; SG—superfine grinding; US—ultrasound; WBM—wet ball milling; HPM—high-pressure microjet); the scale bars are 25 μm.

The ability of proteins to create and stabilize emulsions are essential for many food products, including beverages and condiments. This is due to the fact that most proteins have surface-active properties and can act as surfactants [43]. As shown in Figure 6B, it can be seen that the emulsifying capacity decreased after ball milling and ultrasound treatment. However, it increased to different degree after other physical modifications. It can be observed from the CLSM diagram (Figure 7) that the aggregation of oil droplets occurred in emulsions which used ball milled and ultrasound treated walnut protein and the distribution oil was not uniform. Considering the wet ball milled walnut protein, the emulsion droplets were small and uniform, and there was no obvious agglomeration and flocculation, which confirmed ability of this method to improve emulsifying property of walnut protein. The improvement of solubility walnut protein, particularly by the wet physical method, contributed to the diffusion of walnut protein molecules to the oil-water interface, thus improving the emulsification properties. The surface hydrophobicity of proteins is also considered as a key factor favoring protein-oil interactions [44]. However, excessive hydrophobicity of protein may affect the stable arrangement of particles or molecules on the oil-water interface and make them prone to flocculation [45]. This explained the fact that the high-pressure microjet modified walnut protein had higher surface hydrophobicity than that of wet ball milling modified. However, the emulsion stability of the former was lower.

The ESI of walnut proteins modified by the ball milling, ultrasound, and high-pressure microjet techniques is increased (*p* < 0.05). The ball milling might have improved the ESI due to the unfolding of the WPI and exposure of hydrophobic groups. The ultrasound treatment might have disrupted intermolecular interactions and increased the surface charge of the walnut protein. The increase in negative surface charge (negative zeta potential) of the emulsion droplets provided electrostatic repulsion between the droplets and thus inhibited droplet flocculation and improved emulsion stability [46]. Emulsions stabilized by walnut protein modified through the high-pressure microjet method showed significantly higher stability than those stabilized by walnut protein modified through wet ball milling. This superior stability can be attributed to the increased pressure and rupturing force generated by the high-pressure microjet system, which is expected to facilitate a greater unfolding of walnut protein [47].

## 4. Conclusions

In this work, the effects of physical modification treatments using dry and wet routes were applied to walnut protein to improve its physicochemical and functional properties. The results showed that physical modification had an important effect on the structure, aggregation state, solubility, and other functional properties of walnut protein. High-pressure microjet treatment increased the solubility of walnut protein by 3.6 times, foaming from 47% to 55.33%, and emulsification from 4.32 m^2^/g to 8.27 m^2^/g. These results indicate that high-pressure microjet treatment significantly improved the functional properties of walnut protein. These effects were attributed to cavitation, high shear turbulence, and heating effects prevailing in high-pressure microjet treatment, which led to the partial unfolding of the secondary structure, exposure of hydrophilic groups, and an increase in the negative surface charge of walnut protein, all of which were conducive for improving the solubility and other properties. Wet ball milling improved the foaming and emulsification properties of walnut protein. The wet route-based physical modification was found to be superior to dry route-based physical modification in improving the functional properties of walnut protein. These results will be valuable for industry to produce highly soluble walnut protein-based beverages and emulsions. These findings will contribute to enhancing the value of walnut products and expanding the application of walnut protein.

## Figures and Tables

**Table 1:** Surface hydrophobicity and maximum excitation wavelength of endogenous fluorescence of walnut protein after different physical modifications (Control—control subjects; CP—cold plasma; BM—ball milling; SG—superfine grinding; US—ultrasound; WBM—wet ball milling; HPM—high-pressure microjet); (a–f) represent significant differences between walnut protein at the *p* < 0.05 level.

Samples	H_0_	λ_max_ (nm)
Control	1226.93 ± 16.74 e	347.67 ± 2.39 a
CP	514.39 ± 9.61 f	347.93 ± 0.68 a
BM	1287.37 ± 11.51 d	344.00 ± 0.36 c
SG	548.85 ± 7.41 f	346.47 ± 0.12 b
US	1543.27 ± 6.20 c	343.17 ± 0.85 c
WBM	1867.67 ± 16.11 b	338.50 ± 1.08 d
HPM	3731.57 ± 57.88 a	336.67 ± 0.94 e

The data are expressed as the mean ± standard deviation.

**Table 2:** Secondary structures of walnut protein after physical modifications (Control—control subjects; CP—cold plasma; BM—ball milling; SG—superfine grinding; US—ultrasound; WBM—wet ball milling; HPM—high-pressure microjet); (a–e) represent significant differences between walnut protein at the *p* < 0.05 level.

Samples	α-Helix (%)	β-Sheet (%)	β-Turn (%)	Random Coil (%)
Control	32.63 ± 1.39 b	23.61 ± 3.26 c	20.78 ± 2.58 b	22.99 ± 3.80 a
CP	19.94 ± 1.44 e	37.26 ± 1.14 b	27.91 ± 1.28 a	14.88 ± 0.76 cd
BM	25.18 ± 4.50 cd	34.53 ± 3.25 b	26.64 ± 5.60 a	13.65 ± 0.49 cd
SG	20.13 ± 1.81 e	44.26 ± 3.95 a	18.12 ± 2.72 b	17.48 ± 2.82 bc
US	40.18 ± 2.57 a	22.53 ± 2.71 c	27.17 ± 1.54 a	10.12 ± 2.34 d
WBM	22.88 ± 2.12 de	33.65 ± 1.43 b	21.03 ± 2.06 b	22.43 ± 2.11 ab
HPM	28.13 ± 2.72 c	36.85 ± 1.66 b	19.65 ± 1.22 b	15.36 ± 4.24 cd

The data are expressed as the mean ± standard deviation.

## Data Availability

The datasets generated for this study are available upon request to the corresponding author.
